# Prediction and reasons for COVID-19 second dose vaccine hesitation: a cross-sectional study in a municipality of Brazil

**DOI:** 10.1590/1516-3180.2022.0095.R1.06072022

**Published:** 2022-08-29

**Authors:** Carlos Izaias Sartorão-Filho, Mariana Costa Zoqui, Douglas Otomo Duarte, Edy Alyson Ribeiro, Vinicius César Queiroz Bisetto, Lara Escobar Gavião Cachoni, Ana Luísa Varrone Sartorão, Diogo Coutinho Terribile, Beatriz Balsimelli de Mello, Carlos Izaias Sartorão-Neto, Roberto de Mello

**Affiliations:** I MD, PhD. Professor of Medicine, Fundação Educacional do Município de Assis (FEMA), Assis (SP), Brazil.; II Medicine Student, Fundação Educacional do Município de Assis (FEMA), Assis (SP), Brazil.; III Medicine Student, Fundação Educacional do Município de Assis (FEMA), Assis (SP), Brazil.; IV Medicine Student, Fundação Educacional do Município de Assis (FEMA), Assis (SP), Brazil.; V Medicine Student, Fundação Educacional do Município de Assis (FEMA), Assis (SP), Brazil.; VI Medicine Student, Fundação Educacional do Município de Assis (FEMA), Assis (SP), Brazil.; VII Medicine Student, Fundação Educacional do Município de Assis (FEMA), Assis (SP), Brazil.; VIII Medicine Student, Fundação Educacional do Município de Assis (FEMA), Assis (SP), Brazil.; IX Medicine Student, Fundação Educacional do Município de Assis (FEMA), Assis (SP), Brazil.; X Medicine Student, Faculdade de Medicina da Universidade de São Paulo (FMUSP), São Paulo (SP), Brazil.; XI MD. Deputy Provider, Santa Casa da Misericordia de Assis Assis (SP), Brazil.

**Keywords:** COVID-19, Vaccination hesitancy, Vaccination, Vaccination refusal, Anti-vaccination movement, Coronavirus disease 19, Immunization, active, Anti-vaccine groups

## Abstract

**BACKGROUND:**

Hesitation and refusal to take a second dose of the vaccine for coronavirus disease 19 (COVID-19) are prevalent.

**OBJECTIVES:**

We aimed to identify predictive factors for hesitation or refusal and describe groups with higher rates of vaccine hesitancy.

**DESIGN AND SETTING:**

A cross-sectional study in Assis City, Brazil.

**METHODS:**

The study included adults who passed the due date for taking the COVID-19 second dose vaccine. Participants were recruited in December 2021 using a mobile-based text message. Sociodemographic and clinical data and reasons for hesitance were collected. The outcome was the attitude towards completing the recommended second dose of the vaccine. Bivariate and multivariate Poisson analyses were performed to determine the adjusted predictors.

**RESULTS:**

Participants between 30–44 years of age had a 2.41 times higher prevalence of hesitation than those aged 18–29 years. In addition, people who had adverse events or previously had COVID-19 had 4.7 and 5.4 times higher prevalences of hesitation, respectively (P value < 0.05).

**CONCLUSION:**

We found a significant group of adults aged between 30–44 years who refused the second dose of the COVID-19 vaccine. Furthermore, those who reported adverse effects after the first dose and those who had COVID-19 previously were a significant group for refusal.

## INTRODUCTION

The high morbidity and mortality due to the coronavirus disease 19 (COVID-19) pandemic pose a challenge to health institutions. All governments need to be prepared to ensure large-scale, balanced access and universal distribution of vaccines. This also requires strategies to increase trust in and acceptance of the vaccine by the population that will receive it.^1^

Before the pandemic, the World Health Organization had defined hesitation in taking vaccines as a or refusal or delay in their acceptance, despite the availability of services.^2^ Hesitation can vary in form and intensity according to the place and time in which the vaccine is offered. Concerns about vaccine hesitancy are growing worldwide, with misinformation being a substantial barrier in many countries and constituting an obstacle to immunization and community immune coverage.^2–4^

Moreover, the anti-vaccine movement remains a significant health threat, and new social forces have been set into motion because of COVID-19. The anti-vaccine movement’s new conspiracy theories and alliances have possibly grown stronger worldwide.^5^

Since 2020, after the approval of vaccines for emergency use, most vaccine schemes for COVID-19 have recommended at least two doses at predetermined intervals. However, studies have pointed to a relatively high number of people who do not return for the second dose. In a multicenter study conducted in the United States with 14.2 million people in different locations of the country and different ethnicities the hesitancy rate for the second dose varied between 1–25%.^6^

Currently, there is a great concern about non-acceptance and abstention from taking vaccines made available by government campaigns, including about hesitancy and delay regarding taking the second dose of the vaccine for COVID-19.^7^ In the municipality of Assis, Brazil, official data provided by the Health Department showed that in December 2021 there were 4,667 people with a delay of more than 30 days in taking the second dose of the vaccine, corresponding to approximately 12% of abstention.

## OBJECTIVE

The primary objective of this study was to identify predictive factors for hesitation, delay, or refusal to take a second dose of anti-COVID-19 vaccines by the population of the municipality of Assis.

As specific objectives, we aim to describe groups with higher rates of abstention from the second vaccine dose, and thus contribute to the development and promotion of interventions aimed at reducing rates of hesitation or refusal of the second vaccine dose.

## METHODS

An analytical cross-sectional study was conducted in the Municipality of Assis, Brazil, by the Faculty of Medicine of the Educational Foundation of the Municipality of Assis. Assis is a city in São Paulo state with an estimated population of 105,000 inhabitants and a Human Development Index of 0.805. The study focused on the adult population that passed the due date for taking the second dose of the COVID-19 vaccine. On December 3, 2021, cases with at least 30 days delay in receiving the second vaccine dose were selected from information provided by epidemiological surveillance of the municipality. The study was approved on 29^th^ November 2021, by the Research Ethics Committee (CAAE:51936621.8.0000.8547/Statement:5,135,036).

Eligibility criteria: All adults overdue for more than one month to take the second dose of the COVID-19 vaccine were included; Exclusion criteria: Death from any cause after taking the first vaccine dose.

All participants received an invitation letter via a mobile phone-based text message. Those who consented to participate subsequently received an anonymous, confidential electronic form with questions related to sociodemographic data and the reason for their hesitation in taking the second dose of the vaccine.

We applied questions extracted and adapted to Portuguese from the questionnaire reported by the COVID-SCORE study^8^ regarding vaccine acceptance. Participants answered vaccine safety and efficacy questions and provided age, gender, and education demographic variables.^8^ We also asked about the history of previous COVID-19 infections. The first-dose vaccine’s brand name and date were provided by the census of the epidemiological surveillance of Assis. Finally, participants were asked about the reasons for not having taken the second dose of the vaccine, in a multiple-choice question where they could answer one or more of the following alternatives: difficulty in time or access to the place where the vaccine was administered; forgetfulness; lack of confidence in the vaccine; having had adverse events after the first dose; having had COVID-19 previously; being pregnant; being sick; the desired vaccine was not available; and fear of vaccine side effects.

The outcome was analyzed according to the following question: ‘What is your best guess whether you will take the second dose of the coronavirus vaccine?’ Responses were collected on a five-point Likert psychometric scale: I will take it, I will probably take, no opinion or neutral, I probably will not, I definitely will not.

Age in years was collected and then categorized into 18–24, 25–40, 41–50, 51–60, 61–70, 71–80, and 80 years or older. The patient gender was collected as male, female. Education level was divided into three categories: elementary school, high school, and higher education. The participants were asked if they belonged to the general population or were employed as a healthcare worker. Overall health was self-rated as good or fair/poor, having any comorbidity. We also asked whether the participants reported a history of COVID-19 infection. The data collection period was one week in December 2021. The researchers were responsible for all collection procedures. Text messages were sent twice per week. Data were tabulated and analyzed for comparison between groups and for statistical analysis using bivariate and multivariate Poisson models to meet the objectives proposed by the research. IBM SPSS Statistics for Windows (version 23.0, released in 2015 (Armonk, New York, United States) was used. Categorical variables are presented as frequencies and percentages. Comparisons of these variables were made using the chi-squared test or Fisher’s exact test. The significance level was set at P < 0.05. Crude odds ratios were obtained using bivariate Poisson regression, and the significance level was set at P < 0.20. Adjusted odds ratios considering the significant binary variables for refusing to complete the second vaccine dose were obtained using multivariate Poisson regression, using a significance level of P < 0.05.

The primary outcome was the participants’ preference for not completing the second-dose vaccination. We included those respondents in an ‘indecisive group’ with those who responded that they probably or definitively will not take the second dose.

Sample size calculation: We considered a significance level of 5%, absolute error of 5%, and population size of 3,547 people eligible for the study. The resultant sample size was 347 individuals.

## RESULTS

Official data from the municipality of Assis showed that 4,667 adults had been vaccinated for more than 30 days at the start of the study, totaling 12% abstention. Of these, 908 received the first dose of CoronaVac (Sinovac), 2,075 ChAdOx1 (Oxford–AstraZeneca), and 1,684 BNT162b2 (Pfizer, BioNTech) provided by the Brazilian Ministry of Health since January 2021. Unfortunately, 1,120 records were discarded because of invalid or nonexistent phone numbers. A total of 3,547 participants were selected to receive a text message containing the letter of invitation to participate in the research and in cases of acceptance of the free and informed consent form, the form with the questionnaire via Google Forms. The mean age was 35.05 years (range 18–97 years).

After one week following the invitation 354 people had responded to the questionnaire. An invitation was sent twice per week to each participant. Of the 354 responses, eight were excluded because of reports sent by family members of people who died before taking the second dose of the vaccine. The causes of death were not obtained because this was not the purpose of the study. Table 1 presents the sample profiles of the 346 cases included in the study. Of these, 195 were male and 151 were female. The 18–29, 30–44, 45–59, and over 60 years old groups were represented by 179, 96, 51, and 20 participants, respectively. Regarding education, 80 participants had higher education levels. Forty-seven people self-declared that they had any comorbidities or were older than 60 years. In addition, 36 participants reported having previously had COVID-19. The first dose of vaccine was CoronaVac (Sinovac) in 79 participants, ChAdOx1 (Oxford–AstraZeneca) in 91, and BNT162b2 (Pfizer–BioNTech) in 176. The most common reasons for hesitation to accept the second dose of vaccine were related to difficult access, logistics, or lack of time (56.36%) and forgetting or not paying attention to the delay (24.28%). When asked whether they intended to take the second dose, 285 participants responded positively (82.37% of responses). The delay since the first dose of the vaccine ranged from 30 to 287 days, with a mean of 91.39 days.

**Table 1:** Sample characteristics (n = 346)

		Total	%
**Sociodemographic**			
	**Male (%)**	195	56.36
**Age in years**			
	18–29	179	51.7
	30–44	96	27.7
	45–59	51	14.7
	≥ 60	20	5.8
**Education level**			
	Elementary	97	28.0
	High school	169	48.8
	Higher	80	23.1
	Healthcare worker	9	2.60
**Health status**			
	Self-rated comorbidities or elder	47	13.58
	Belongs to general population	294	84.97
	Previous COVID-19	36	10.40
**First dose vaccine**			
	CoronaVac (Sinovac)	79	22.83
	ChAdOx1 (Oxford–AstraZeneca)	91	26.30
	BNT162b2 (Pfizer–BioNTech)	176	50.87
**Reasons for the hesitance**			
	Logistics, time or difficult access	195	56.36
	Forgetfulness	84	24.28
	Lack of confidence in the vaccine	10	2.89
	Had adverse events after the first dose	7	2.02
	Previous COVID-19 and consider yourself immune	2	0.58
	Pregnancy	3	0.87
	Sickness	14	4.05
	There was no vaccine of the desired brand	5	1.45
	Afraid of vaccine side effects	30	8.67
	Preferred not to answer	9	2.60
**Attitude towards completing the vaccine scheme**			
	I will take it	262	75.72
	Probably will take it	23	6.65
	Indifferent or indecisive	16	4.62
	Probably will not take it	8	2.31
	Definitely will not take it	13	3.76
	Prefer not to answer	23	6.65
	Tendency to refuse vaccination	60	17.34

COVID-19 = coronavirus disease 2019.

The associations of the variables in Table 1 with participants’ refusal to take the second dose of the vaccine were subjected to bivariate analysis. Participants aged 30–44 years, with higher education, comorbidities or advanced age, a declared lack of confidence in vaccines, already had COVID-19, afraid of the vaccine, and adverse side effects after taking the first dose were identified in the bivariate association (P < 0.20) as most likely to refuse to complete the vaccination schedule (Table 2).

**Table 2:** Bivariate Poisson distribution. Associations between sociodemographic variables, health status, and reasons for not taking the second dose with refusal to take the second dose. (n = 346)

Variable		Second dose refusal				Total	P
		Not		Yes			
		n	%	n	%	n	
**Sociodemographic**							
**Gender**							
	Female	124	82.1	27	17.9	151	1.000
	Male	161	82.6	34	17.4	195	
**Age in years**							
	18–29	158	88.3	21	11.7	179	0.002
	30–44	67	69.8	29	30.2	96	
	45–59	44	86.3	7	13.7	51	
	≥ 60	16	80.0	4	20.0	20	
**Education**							
	Elementary	79	81.4	18	18.6	97	0.037
	High	147	87.0	22	13.0	169	
	Higher	59	73.8	21	26.3	80	
**Health worker**							
	No	277	82.2	60	17.8	337	0.001
	Yes	8	88.9	1	11.1	9	
**Belongs to the general population**							
	No	40	76.9	12	23.1	52	0.322
	Yes	245	83.3	49	16.7	294	
**Self-rated overall health**							
**Comorbidities or elder**							
	No	250	83.6	49	16.4	299	0.148
	Yes	35	74.5	12	25.5	47	
**First dose vaccine brand**							
**CoronaVac (Sinovac)**							
	No	220	82.4	47	17.6	267	1.000
	Yes	65	82.3	14	17.7	79	
**ChAdOx1 (Oxford–AstraZeneca)**							
	No	208	81.6	47	18.4	255	0.529
	Yes	77	84.6	14	15.4	91	
**BNT162b2 (Pfizer–BioNTech)**							
	No	142	83.5	28	16.5	170	0.672
	Yes	143	81.3	33	18.8	176	
**Previous COVID-19**							
	No	258	83.2	52	16.8	310	0.247
	Yes	27	75.0	9	25.0	36	
**Reasons for the hesitance/delay**							
**Logistic, lack of time or access**							
	No	102	67.5	49	32.5	151	0.001
	Yes	183	93.8	12	6.2	195	
**Forgetfulness**							
	No	205	78.2	57	21.8	262	0.001
	Yes	80	95.2	4	4.8	84	
**Lack of confidence**							
	No	283	84.2	53	15.8	336	0.001
	Yes	2	20.0	8	80.0	10	
**Adverse events in the first dose**							
	No	284	83.8	55	16.2	339	0.001
	Yes	1	14.3	6	85.7	7	
**Due to prior COVID-19**							
	No	285	82.8	59	17.2	344	0.031
	Yes	0	0.0	2	100.0	2	
**Pregnancy**							
	No	283	82.5	60	17.5	343	0.442
	Yes	2	66.7	1	33.3	3	
**Sickness**							
	No	275	82.8	57	17.2	332	0.282
	Yes	10	71.4	4	28.6	14	
**Not desired vaccine brand**							
	No	282	82.7	59	17.3	341	0.214
	Yes	3	60.0	2	40.0	5	
**Afraid of side effects**							
	No	275	87.0	41	13.0	316	0.001
	Yes	10	33.3	20	66.7	30	
**Did not answer**							
	No	285	84.6	52	15.4	337	0.001
	Yes	0	0.0	9	100.0	9	

Chi-square and Fisher’s exact test. P-value < 0.20; COVID-19 coronavirus disease 2019.

Based on the results shown in Table 2, we developed multivariate Poisson models to explain the prevalence of refusal as a function of the following variables: age, education, having comorbidity or being elderly, not having confidence in the vaccine, having had an adverse event with the first dose, having already had COVID-19, and being afraid of side effects. We assessed the association between the independent variables using the homoscedasticity test (Table 3).

**Table 3:** Homoscedasticity test for the independent variables

	Higher-level	Comorbidities	Lack of confidence	Previous adverse effects	Previous COVID-19	Afraid of collateral effects
Age	0.001	0.001	0.003	0.329	0.001	0.001
Educational level		0.214	0.842	0.534	0.487	0.001
Comorbidities			0.142	1.000	1.000	0.781
Lack of confidence				1.000	1.000	0.007
Previous adverse effects					1.000	0.473
Previous COVID-19						1.000

P < 0.05; COVID-19 = coronavírus disease 2019.

We observed heterogeneity of variance in the following variables: education, comorbidities, not feeling confident, and fear of side effects; these can be explained by age so they were not included in the model. Thus, model proposals included the variables with homoscedasticity: age, already had an adverse event, and already had COVID-19, as shown in Table 4.

**Table 4:** Multivariate Poisson Models adjustment to explain the vaccine’s second dose’s refusal

Variable		PR	95% CI		P
≥ 60 years		1.56	0.53	4.58	0.416
45–59 years		1.27	0.54	3.00	0.583
30–44 years		2.41	1.37	4.23	0.002
**Age (Reference: 18–29 years)**					
	Adverse events after the first dose	4.72	2.00	11.11	0.001
	Previous COVID-19	5.44	1.31	22.49	0.019

P < 0.05; PR = prevalence risk; CI = confidence interval; COVID-19 = coronavirus disease 2019.

According to the adjusted model people between 30 and 44 years of age had a 2.41 times higher prevalence of hesitation in taking the second dose than people between 18 and 29 years of age. People who had adverse events had a 4.7 times higher prevalence of hesitation in taking the second dose, and people who had already had COVID-19 had a 5.4 times higher prevalence of hesitation in taking the second dose (P value < 0.05).

## DISCUSSION

In the city of Assis, Brazil, where 12% of the adult population was more than 30 days later for the second dose of the vaccine for COVID-19, we identified that the independent variables that were most associated with increased risk were the population aged 30–44 years, those who experienced adverse effects after the first dose, and those who had previously had COVID-19. Vaccine hesitancy is a worldwide phenomenon, with a variety of reasons for refusing to accept the vaccine. Common reasons include perceived risks versus benefits, religious beliefs, and lack of knowledge and awareness.^8,9^ In this study we found that the main reasons for not returning to take the second dose were forgetfulness, difficulties accessing the site, or lack of time to get vaccinated. Despite the full and free availability of vaccines in the municipality, we believe that logistic reasons were associated with socioeconomic status. Studies suggest that public misinformation about COVID-19 may be contributing to hesitancy to get the vaccine.^10^ The findings highlight the need for measures to address public acceptability, such as measures to increase trust and reduce concern about the safety and benefit of approved vaccines.^11^ A study conducted in Ghana, Africa, reported that participants 36–45 years old were the most hesitant, similarly to our study results.^12^

In an online survey in Brazil with 173,178 respondents the overall vaccine hesitancy was 10.5%, similar to our study (12%) and this was considered low compared to other countries.^13^

Although the results of other studies provided empirical support for the benefits of being able to choose a vaccine in increasing willingness,^14^ our study did not show significant differences among the associations of the three varieties of vaccines used in the municipality with refusal or hesitance.

There was high vaccine acceptability among health care professionals, with only nine from more than 2,000 with this profile who did not take the second dose of the vaccine. This result was similar to that of a study in Mozambique, where the acceptability of the vaccine was 86.6% among health professionals.^15^

Study limitations: Little information exists in the literature to explain why adults aged between 30 and 44 refuse the vaccine. In addition, cross-sectional studies are susceptible to research bias owing to inadequate responses and possible misclassifications. We used a questionnaire based on COVID-SCORE questions;^8^ however, it was not validated in Portuguese. Furthermore, we did not assess the associations of ethnicity, marital status, political view, or socioeconomic status with vaccine hesitancy.

In our study the response rate to a mobile phone-based text message was 9.99%. In addition, we have already published a secondary study concerning the representativeness and efficacy of the phone-based text message survey in a population that hesitated in taking the COVID-19 vaccination.^16^ The mean age of the respondent group was 33.97 (standard deviation 14.99). In comparison with the characteristics of our eligible population, Cohen’s d coefficient was 0.0754, corresponding to a small effect size between the respondents and the eligible population as a reference. Thus, we suppose that a mobile phone-based survey is a feasible and representative strategy during the pandemic in Brazil.

Moreover, older respondents were representative.^16^ Furthermore, we received feedback with commentaries from several respondents, most of whom requested further information on the vaccination campaign and acknowledging the advice to complete the proposed scheme. Thus, we consider strengths of our study to be not only the survey strategy, but also the acceptance of the population after receiving the text message and self-reported commitment to get more orientation regarding the vaccination.

Despite the parochial context of our study, we believe that our findings can be generalized to other populations nationwide and worldwide, particularly to low-income populations.

## CONCLUSION

We found a significant group of adults between 30 and 44 years of age, intending to refuse the second dose of the COVID-19 vaccine. Furthermore, those who experienced adverse effects after the first dose and those who had COVID-19 previously were representative and independent groups for refusing the second dose.

The scientific community must detect the reasons for hesitation or non-acceptance of vaccines and focus on these predictor risk groups when developing campaigns to raise awareness about the benefits of adequate immunization for the population.
